# Historical and future maximum sea surface temperatures

**DOI:** 10.1126/sciadv.adj5569

**Published:** 2024-01-26

**Authors:** B. B. Cael, Friedrich A. Burger, Stephanie A. Henson, Gregory L. Britten, Thomas L. Frölicher

**Affiliations:** ^1^ National Oceanography Centre, Southampton, UK.; ^2^ Climate and Environmental Physics, Physics Institute, University of Bern, Bern, Switzerland.; ^3^ Oeschger Centre for Climate Change Research, University of Bern, Bern, Switzerland.; ^4^ Woods Hole Oceanographic Institution, Woods Hole, MA, USA.; ^5^ Massachusetts Institute of Technology, Cambridge, MA, USA.

## Abstract

Marine heat waves affect ocean ecosystems and are expected to become more frequent and intense. Earth system models’ ability to reproduce extreme ocean temperature statistics has not been tested quantitatively, making the reliability of their future projections of marine heat waves uncertain. We demonstrate that annual maxima of detrended anomalies in daily mean sea surface temperatures (SSTs) over 39 years of global satellite observations are described excellently by the generalized extreme value distribution. If models can reproduce the observed distribution of SST extremes, this increases confidence in their marine heat wave projections. 14 CMIP6 models' historical realizations reproduce the satellite-based distribution and its parameters’ spatial patterns. We find that maximum ocean temperatures will become warmer (by 1.07° ± 0.17°C under 2°C warming and 2.04° ± 0.18°C under 3.2°C warming). These changes are mainly due to mean SST increases, slightly reinforced by SST seasonality increases. Our study quantifies ocean temperature extremes and gives confidence to model projections of marine heat waves.

## Teaser

CMIP6 models capture the observed distribution of maximum sea surface temperatures, supporting projections of marine heat waves.

## INTRODUCTION

Marine heat waves (MHWs)—anomalously high ocean temperatures (*1*)—can extend thousands of kilometers and last for weeks to years (*2*, *3*). MHWs have occurred in all ocean basins over the past few decades (*4*, *5*) and often caused devastating impacts on marine ecosystems (*6*), ranging from habitat shifts (*7*) and changes in population structure (*8*) to high mortality of various marine keystone species (*9*, *10*). These extreme events can overwhelm the capacity of both natural and human systems to cope, potentially causing socioeconomic impacts such as loss of essential ecosystem services and fisheries income (*6*, *11*). The frequency of MHWs has increased over the past century (*12*), including a doubling over the satellite period (*4*), mainly due to anthropogenic climate change (*3*, *4*). The frequency and intensity of MHWs are projected to increase in the future as global temperatures are projected to continue to rise (*4*, *5*) with potentially widespread consequences for marine ecosystems globally. However, the reliability of these projections is uncertain because the models used to make them have not been statistically compared to historical observations of MHWs. In this study, we test these models’ ability to capture the observed statistics of maximum ocean temperatures to evaluate how reliable their future projections of MHWs may be.

To do so, we use the generalized extreme value (GEV) distribution, a well-established statistical model to describe the maxima of temperature distributions (or maxima of any other time series data) (*13*). The GEV distribution has been applied to study, for example, extreme temperatures and precipitation on land (*14*–*18*). While there has been some application of the GEV in marine contexts (*19*, *20*), it remains underused in oceanic applications and, in particular, in studies of MHWs (*21*).

Analogous to the Gaussian distribution and the central limit theorem (*22*), the maxima of many natural phenomena are GEV-distributed, explained by the extreme value theorem (*13*). The GEV distribution’s three parameters, location [μ, (°C)], scale [σ, (°C)], and shape (ξ), roughly determine its central value, its variability, and the weight of its upper tail (Materials and Methods). The advantage of a distributional approach is that if the GEV can describe the variability in observation-based sea surface temperature (SST, °C) maxima, this simplifies the description and quantitative comparison with climate models. The question of how statistically similar models and observations’ SST maxima are becomes a question of how GEV-like modeled and observed SST maxima are, what the parameters of the associated distributions are, and how these parameters vary in space when estimated for individual locations.

Our analysis starts with the hypothesis that SST maxima are GEV-distributed. Here, we test this hypothesis for satellite-derived annual maxima of mean daily SST. We then test whether SSTs simulated by the latest generation of Earth system models that participated in phase 6 of the Coupled Model Intercomparison Project [CMIP6; (*23*)] capture the statistical characteristics of observed SST extremes well. We then use this finding to make inferences about future ocean temperature extremes under two different global warming scenarios.

## RESULTS

**Fig. 1.**
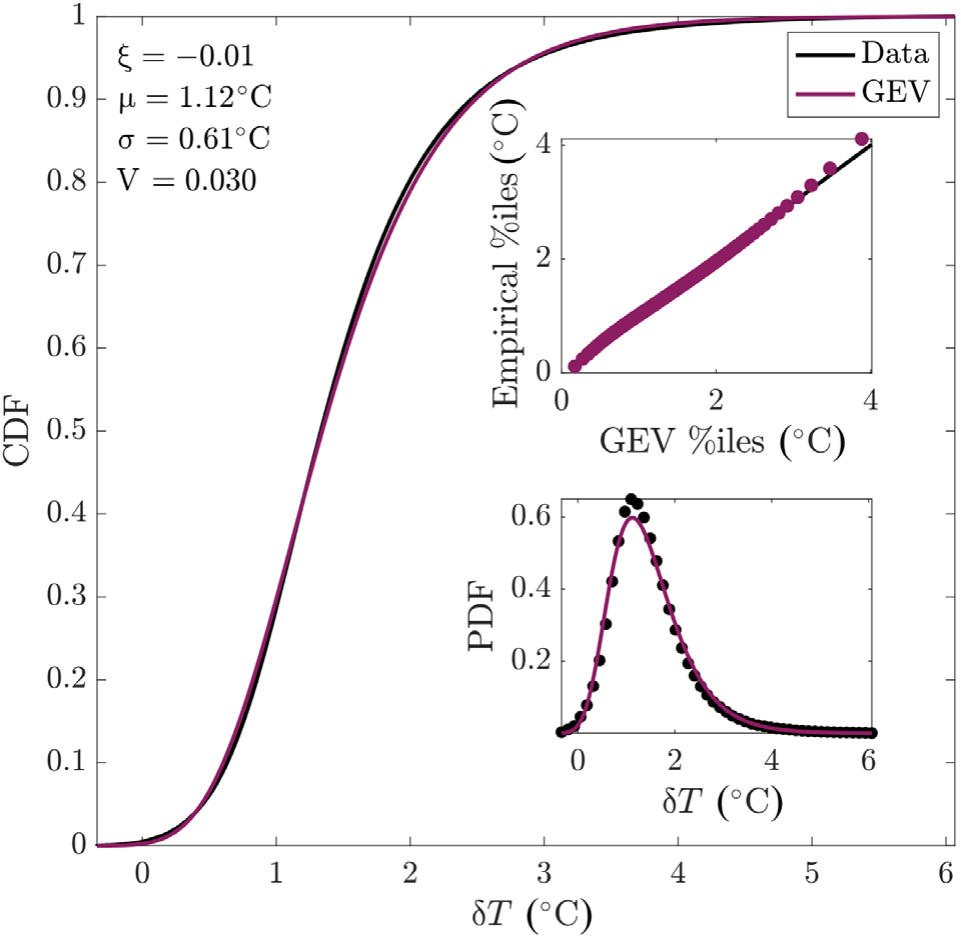
GEV distribution fit for globally pooled maximum annual SST anomalies. Shown are the empirical and GEV (fit to the observations) cumulative distribution function (CDF), with the corresponding empirical and GEV probability density function (PDF; as a histogram for the empirical PDF) in the bottom inset, and in the top inset the empirical versus GEV percentiles (%iles; i.e., the percentiles of the observations and the fitted GEV, respectively) overlaid on a 1:1 line. The fit parameters for shape (ξ), location [μ, (°C)], scale [σ, (°C)], and the Kuiper statistic (*V*) are given. Data are analyzed at 1° resolution to facilitate comparison with models. δ*T* (°C) is the annual maximum daily mean SST anomaly after removing the interdecadal trend and seasonal cycle (Materials and Methods). Note that by construction, the CDF plots each observational value individually, in ranked order, whereas the inset figures plot different simplified approximations of this distribution. The time period analyzed is 1982–2020.

We find that the GEV is appropriate for modeling annual maxima in SST (Fig. 1). When pooling all annual maxima of linearly detrended SST anomalies over the 39-year satellite-based observation period (1982 to 2020) over all grid cells across the globe (see Materials and Methods), the GEV distribution captures the shape of the empirical distribution excellently. This is seen visually in Fig. 1 and quantified by the Kuiper statistic *V*, which measures the difference between two distributions in terms of the maximum differences in their cumulative distribution functions (CDFs) (Materials and Methods). The Kuiper statistic is similar to the more common Kolmogorov-Smirnov statistic but is preferred because it is equally sensitive for all SST values (*24*). No significant trends in the parameter estimates can be found over the 39-year period; specifically, we repeated the analysis shown in Fig. 1 for individual years, both globally and regionally, and found no significant trends in the estimated GEV parameters (Materials and Methods). The parameter estimates of distributions for individual years do not change systematically with time. Hence, we do not find evidence of nonstationarity in the distribution of annual maxima of detrended SST anomalies.

At the local scale, the GEV is fitted to detrended SST anomalies as well as to raw SST data (see Materials and Methods). The goodness of fit is assessed on the basis of the median Kuiper statistic; we find a median Kuiper statistic of 0.14 (anomalies) and 0.13 (raw data). In the ideal case of sampling 39 values from a GEV distribution many times, one also obtains a very similar median Kuiper statistic of 0.14 (Materials and Methods), suggesting that the GEV is a good model also at the local scale. In other words, a Kuiper statistic value of 0.14 is expected for true GEV data given the sample size, which matches the values found for the observations. In Materials and Methods, we also describe a sensitivity test showing that the GEV is applicable at the regional (10° × 10°) scale, in addition to the local (1° × 1°) and global scales.

**Fig. 2.**
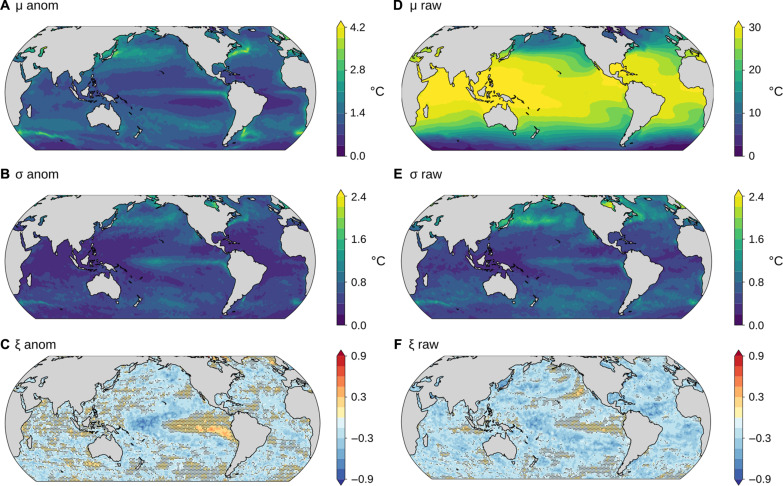
Local GEV parameter estimates for the satellite SST observations. Estimated parameters are shown for the anomalies (first row) and raw data (second row). Black stippling in (**C** and **F**) indicates regions where the estimate’s 90% confidence interval includes 0; no such region exists for (**A**, **B**, **D**, or **E**). μ (°C), σ (°C), and ξ are the location, scale, and shape parameters of the GEV distribution fit to raw or anomaly SST maxima at each location (Materials and Methods). “raw” refers to the annual maximum daily SSTs, and “anom” refers to the anomalies of these relative to an interdecadal trend and seasonal cycle (Materials and Methods). The color scale is different for each subfigure. The time period analyzed is 1982–2020.

The spatial pattern in the location parameter for the raw data (Fig. 2D) mainly reflects the latitudinal gradients in SSTs, with higher maxima in low-latitude regions where SST is generally higher. For the detrended anomaly data (Fig. 2A), we find the largest location parameters where SST variability is largest, such as in western boundary current regions (*25*) and the high latitudes (*26*). The scale parameter is generally large where strong interannual variability in SST drives large year-to-year variations in SST maxima (Fig. 2, B and E), such as in the equatorial Pacific and in the northern high latitudes. The scale parameter estimates are often larger for the raw data (median ratio σ anom./σ raw = 0.79, 90% range 0.56 to 1.16) because detrending reduces the year-to-year variability in the SST maxima relative to the raw SST data (see Materials and Methods for uncertainties). The shape parameter is close to zero over much of the ocean (Fig. 2, C and F) and slightly negative elsewhere. The mean and SD of the shape parameter fit to local anomaly maxima (Fig. 2C) are −0.15 and 0.17, generally consistent with the value of ξ = −0.01 for a GEV fit to the global distribution of anomaly maxima (Fig. 1).

There are no systematic deviations between the CMIP6 Earth system model ensemble and the satellite observations (Table 1). For the globally pooled data, the goodness of fit matches that of the satellite observations well (model-mean Kuiper statistic of 0.032 compared to 0.030 for the satellite data; Table 1). The model-mean parameter estimates are relatively close to the estimates of the satellite product. The observations easily fall within the 90% confidence interval of the model ensemble for every parameter. The satellite-data parameter estimates are thus not significantly different from the respective model distributions. Put differently, the satellite data are indistinguishable from being another model in the CMIP6 model ensemble. While we do not find a systematic difference between the model parameter estimates and those from satellite data, there is notable variation within the model parameter estimates. For instance, the global location and scale parameter estimates range from 0.72° to 1.37°C and 0.49° to 0.76°C, respectively (Table 1). However, analysis of an ensemble of realizations from a single model (see below) suggests that internal variability may explain two-thirds of the multimodel variation for σ and half of that for μ.

**Table 1.** GEV distribution fits for the satellite observations and CMIP6 models’ historical simulations. For the globally pooled anomalies, the Kuiper statistic (*V*) as well as the parameter estimates are shown. For the fits at each location using anomalies and raw data, the median Kuiper statistic ($\tilde{V}$) as well as *r*^2^ values for the simulated μ and σ parameters are shown, indicating how well the simulated parameter estimates from the models’ historical runs agree with those from the observations (see Materials and Methods). An *r*^2^ value of 1 indicates an everywhere perfect match between the parameter estimates in a simulation and those from observations. For this comparison, the models’ historical runs were treated identically to the observations in terms of detrending and deseasonalizing (Materials and Methods). The last two table rows give the ensemble mean and 90% confidence interval (CI) width of the GFDL ESM2M-LE single-model 30-member ensemble. The time period analyzed is 1982–2020.

	Global				Anomalies			Raw		
	*V*	μ	σ	ξ	$\tilde{V}$	*r*^2^ (μ)	*r*^2^ (σ)	$\tilde{V}$	*r*^2^ (μ)	*r*^2^ (σ)
Observations	0.030	1.12	0.62	−0.01	0.14	–	–	0.1	–	–
ACCESS-CM2	0.020	0.83	0.54	−0.03	0.14	0.87	0.87	0.14	0.99	0.90
ACCESS-ESM1-5	0.030	0.72	0.51	−0.01	0.14	0.83	0.87	0.14	0.99	0.89
BCC-CSM2-MR	0.036	0.77	0.49	−0.04	0.14	0.84	0.87	0.14	0.99	0.92
CanESM5	0.022	0.89	0.56	−0.01	0.14	0.82	0.77	0.15	0.99	0.75
CMCC-ESM2	0.038	1.02	0.74	−0.06	0.14	0.91	0.81	0.14	0.99	0.87
CNRM-CM6-1	0.023	1.37	0.62	+0.03	0.14	0.88	0.86	0.14	0.97	0.89
CNRM-ESM2-1	0.029	1.34	0.65	+0.05	0.14	0.81	0.81	0.14	0.98	0.86
CESM2	0.043	0.76	0.56	−0.08	0.14	0.88	0.88	0.14	0.99	0.87
GFDL-ESM4	0.018	1.30	0.64	−0.08	0.14	0.92	0.90	0.14	0.98	0.91
MIROC6	0.030	0.75	0.58	−0.04	0.13	0.82	0.85	0.14	0.96	0.89
MPI-ESM1-2-HR	0.037	0.90	0.55	−0.06	0.14	0.90	0.89	0.14	0.97	0.91
MPI-ESM1-2-LR	0.043	0.85	0.53	−0.07	0.14	0.87	0.90	0.14	0.99	0.91
NorESM2-LM	0.034	1.06	0.66	−0.06	0.14	0.91	0.86	0.14	0.98	0.83
NorESM2-MM	0.056	0.95	0.76	−0.09	0.14	0.991	0.77	0.14	0.98	0.83
Model mean	0.032	0.97	0.60	−0.04	0.14	0.87	0.85	0.14	0.98	0.87
Model 90% CI	±0.018	±0.36	±0.13	±0.07	±0.00	±0.06	±0.07	±0.00	0.02	±0.07
GFDL ESM2M-LE mean	0.033	0.97	0.54	−0.05	0.14	0.83	0.80	0.14	0.99	0.79
GFDL ESM2M-LE 90% CI	±0.023	±0.19	±0.09	±0.09	±0.00	±0.00	±0.02	±0.00	±0.00	±0.02

At the local scale, the models show a very similar goodness of fit as the satellite observations (median Kuiper statistic in Table 1). Furthermore, the parameter estimates agree well with those of the satellite data. The *r*^2^ values for μ and σ, which express the proportions of variance in the model estimates that can be accounted for by the satellite estimates, are often close to 0.9 or higher (Table 1; Materials and Methods). The best match is found for the raw μ estimates because the models and satellite observations generally agree on the latitudinal temperature gradient that imprints on μ for the raw data.

We further support these results by repeating the analysis in Table 1 for the 14-model CMIP6 analysis with an ensemble of 30 realizations with a single model from CMIP5, GFDL ESM2M-LE (Materials and Methods) (*27*). This allows us to test the extent to which differences between models’ values in Table 1 are due to structural differences between models versus internal variability. The intramodel spread (calculated as the width of the 90% confidence interval) for this single-model ensemble in the global *V* and ξ values is larger than that for the 14-model CMIP6 ensemble; that of the global σ parameter is similar in magnitude. This suggests that variations in these parameters across models are dominated by internal variability, further supporting the conclusion that the observed behavior of SST maxima is not distinguishable from models. The CMIP6 intermodel spread is appreciably larger than that of the GFDL ESM2M-LE intramodel spread for the global μ parameter, the *r*^2^ of the model versus observational local σ parameters, and the *r*^2^ of the model versus observational local μ parameter for the SST anomaly maxima. This suggests that models differ in their ability to capture the mean intensity and spatial patterns of SST maxima, which could be leveraged to make constrained projections of future SST maxima. Here, we instead take a more conservative approach, considering future changes in SST maxima only where (i) the CMIP6 14 model ensemble’s 90% confidence interval for the change of a given quantity excludes zero, and also (ii) the CMIP6 14 model ensemble’s 90% confidence interval for that quantity itself includes the observational value. That is, we look for changes where the observed value of a quantity falls within the models’ range of values and where the models agree on the sign of change of that quantity.

**Fig. 3.**
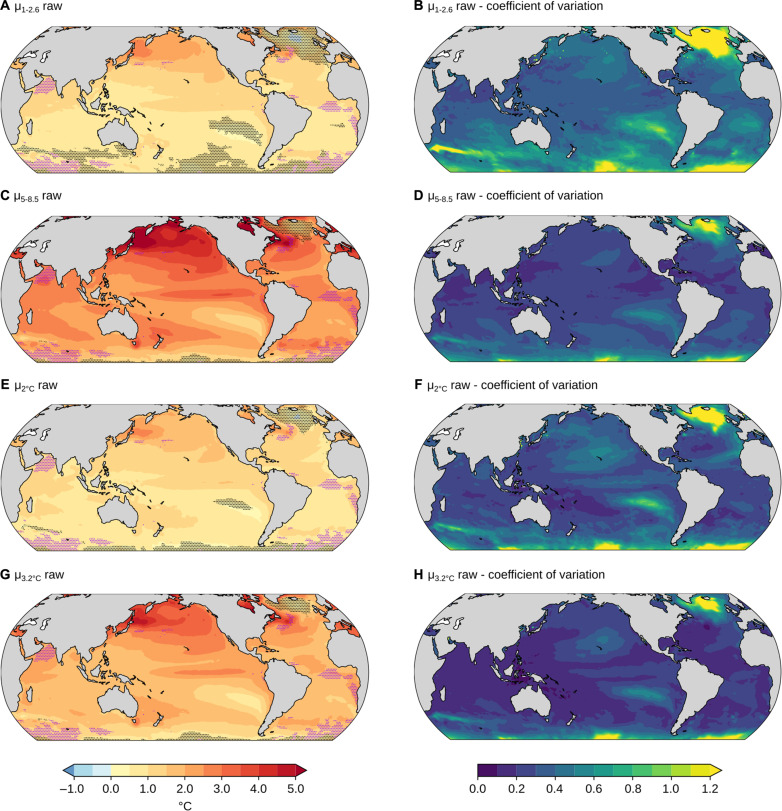
CMIP6 changes in μ. Ensemble mean (**A**, **C**, **E**, and **G**) and coefficient of variation (**B**, **D**, **F**, and **H**) of the change in location parameter μ for raw SST data between the 1982–2020 satellite period and [(A) and (B)] 2061–2100 under the SSP1-2.6 scenario, [(C) and (D)] 2061–2100 under the SSP5-8.5 scenario, [(E) and (F)] the 40-year period centered on the 2°C warming level in the SSP5-8.5 scenario, and [(G) and (H)] the 40-year period centered on the 3.2°C warming level in the SSP5-8.5 scenario. Black stippling in (A) (C), (E), and (G) indicates regions where the 90% confidence interval of the model ensemble distribution includes 0, i.e., that a parameter change of 0 cannot be rejected based on the model ensemble distribution or, equivalently, that the coefficient of variation is >0.61. Pink stippling indicates regions where the parameter estimate from satellite observations is not contained in the 90% confidence interval of the model ensemble distribution during the 39-year historical period. In these regions, the observed GEV distribution thus significantly differs from the models, and it cannot be expected that the future parameter change can be represented by the model ensemble distribution.

**Fig. 4.**
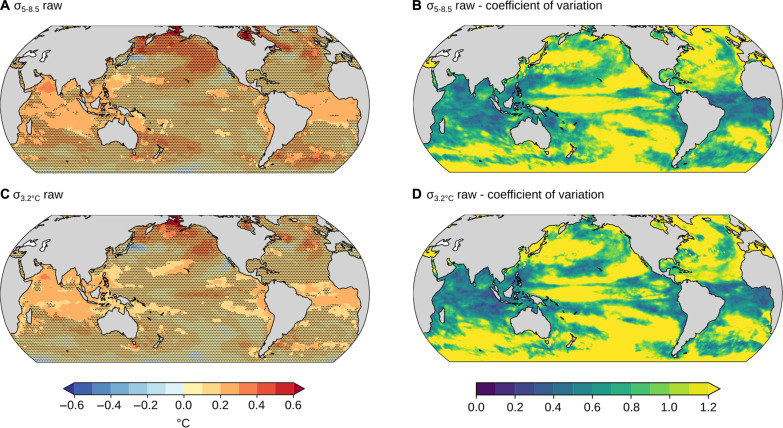
CMIP6 changes in σ. Ensemble mean (**A** and **C**) and coefficient of variation (**B** and **D**) of the change in the scale parameter σ between the satellite period and [(A) and (B)] 2061–2100 under the SSP5-8.5 scenario or [(C) and (D)] after 3.2°C global warming. As Fig. 3 but for σ.

**Fig. 5.**
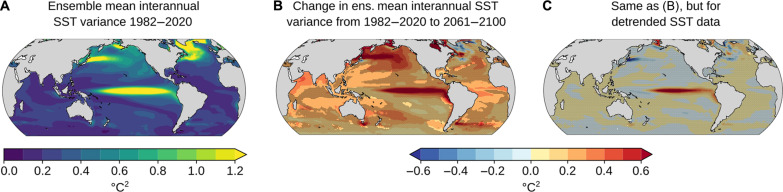
CMIP6 ensemble mean interannual SST variance. (**A**) Ensemble mean SST variance 1982–2020. (**B**) Difference in ensemble mean interannual SST variance 1982–2020 versus 2061–2100 under SSP5-8.5. (**C**) Same as (B) but when annual mean SSTs are (linearly) detrended. Black stippling in (B) and (C) indicates regions where the 90% confidence interval of the model ensemble distribution includes 0, i.e., that interannual SST variance change of 0 cannot be rejected based on the model ensemble distribution.

Where satellite observations fall within the spread of model results in the 39-year historical period (all ocean area outside the pink stippled areas in Fig. 3), one may also expect that the spread of projected changes in GEV parameters with global warming contains the “true” change in parameters under a forcing scenario. We here focus on the location and scale parameters for the raw data, μ_raw_ and σ_raw_. For the other cases (ξ_raw_, μ_anom._, σ_anom._, and ξ_anom._), the models generally do not predict substantial changes nor agree on the sign of change, i.e., the 90% confidence intervals there include zero over almost all of the ocean. The location parameter for the raw SST data increases almost everywhere between the observation period 1982–2020 and 2061–2100, both under SSP1-2.6 and SSP5-8.5 (Fig. 3, A and C). This increase is mainly due to the mean sea surface warming that is simulated by all models in most regions. Exceptions are parts of the Southern Ocean and the North Atlantic, where trends in SST are not always positive (black stippled regions in Fig. 3) (*28*–*30*). Increases in the location parameter μ are generally larger under SSP5-8.5 than under SSP1-2.6, reflecting the larger warming under higher radiative forcing in SSP5-8.5 (Fig. 3). Across all models and over the total ocean, the average difference in μ under SSP5-8.5 versus SSP1-2.6 in 2061–2100 is 1.24°C. Robust increases in σ are simulated for the raw data in the tropical Atlantic and Indian Ocean under SSP5-8.5 scenario (Fig. 4), but not for SSP1-2.6 (not shown in figure). The increases under SSP5-8.5 may simply occur because of increasing warming trends with respect to the period 1982–2020, artificially increasing the interannual variability (*31*) and scale parameter of the GEV distribution. To investigate whether these significant changes in Fig. 4 were due to changes in mean SST trends or to changes in interannual variability, Fig. 5 shows the ensemble mean interannual SST variance, its change from 1982 to 2020 versus 2061 to 2100, and its change from 1982 to 2020 versus 2061 to 2100 after detrending. Significant increases in the σ parameter are mainly simulated in tropical regions of the Indian Ocean and Atlantic Ocean, where interannual variability is generally low (Fig. 5A). It is also in these regions where significant increases in interannual variability are simulated (Fig. 5B), linking changes in interannual variability and σ. Last, no significant increases in interannual variability or σ are found when detrending the data (Fig. 5C), suggesting that the apparent increase in σ in the raw data in those regions is due to increasing warming trends in the SSP5-8.5 simulations, as in (*4*, *31*).

When using fixed warming levels of 2° and 3.2°C instead of a fixed future period, regions where the model ensemble distribution includes zero are similar (black stippling areas in Fig. 3; 3.2°C is used as it is the maximum warming level possible to analyze given the warming in the model realizations investigated here). Furthermore, the coefficient of variation is not substantially reduced by considering warming level rather than time period. The median ratio between the coefficient of variation in μ in the 3.2°C warming level case (Fig. 3H) versus in the end-of-century SSP5-8.5 case (Fig. 3D) is 0.81. This suggests that only ∼20% of the intermodel disagreement in μ changes is due to the CMIP6 models’ different warming rates. Thus, the disagreement between models in these regions is not primarily caused by differing warming rates between the models. The global average increase in the GEV-based expected value of SST maxima is 1.07° ± 0.17°C (mean and SD across models) under 2°C warming and 2.04° ± 0.18°C under 3.2°C warming. These changes are almost entirely (>95%) due to changes in μ, noting that all three parameters can affect the expected value of the GEV. This is slightly greater than the global mean SST increase in these models, which increase by 0.91° ± 0.15°C and 1.76° ± 0.14°C on average in the 2° and 3.2°C cases, respectively, consistent with previous work (*4*). The larger increase in annual SST maxima than in mean SST is almost entirely because of increasing seasonal cycle amplitudes (*32*, *33*). An increase in SST seasonality with ocean warming is caused by larger increases in ocean surface temperature in summer compared to winter that result from greater warming from air-sea heat fluxes in summer when surface mixed layers are shallower (*32*). Increases in seasonal cycle amplitude, i.e., the difference between maximum and minimum of the mean seasonal cycle over an analyzed period, are simulated by all models between the 1982–2020 period and the 40-year periods corresponding to the 2° and 3.2°C warming levels. Consequently, the difference between the maximum of the seasonal cycle and annual mean conditions also increases. On average, across the model ensemble, the difference between SST seasonal cycle maximum and mean SST increases 0.14°C between the 1982–2020 period and 2°C warming period, and 0.25°C for the 3.2°C warming period. Thus, increases in seasonality are responsible for 13% (0.14 of 1.07°C) and 12% (0.25 of 2.04°C) of total increases in annual SST maxima, respectively, in each case.

## DISCUSSION

Our results demonstrate the utility of the generalized extreme value distribution for investigating extreme ocean surface temperatures. We find almost no evidence for heavier tails of maximum SSTs than that of the Gumbel (ξ = 0 case) distribution (i.e., almost no evidence that ξ > 0, where a more positive ξ value is associated with a higher probability of “extreme extremes” in SST). This is to some extent expected because there are numerous stabilizing feedback processes for SSTs, including exchange with the atmosphere and both vertical and lateral mixing. It may also be because we analyze the observations at 1° resolution to facilitate comparison with models as spatial averaging necessarily truncates the tails of temperature maxima. Note that the GEV distribution’s parameters’ values depend on the block size considered and, relatedly, the spatial and temporal scales used. The important aspects of our analysis are therefore that the GEV is a good descriptor of SST maxima and that models and observations yield similar parameter values and spatial patterns at a given scale. It will be valuable in future work to further explore the dependency of GEV parameters on the spatial scale of analysis, particularly with respect to ξ. That said, extreme temperature phenomena in the ocean occurring on larger scales (i.e., >1°) may be of greater interest due to their larger potential impacts, although the larger the spatial scale investigated, the less representative the average is of conditions experienced at a given location. We also find no evidence for nonstationarity in the detrended and deseasonalized SST anomalies, i.e., changes in the distribution of extremes over the 39-year historical period, although this may be due to small sample size and may be detectable in future work via large ensembles of historical simulations (*34*).

Maximum SST is connected to MHWs as it reflects the intensity of the strongest MHW in a year, either for summertime MHWs (that is hottest day of the year for the raw data) or for MHWs occurring throughout the year (largest warm anomalies for the deseasonalized data). Similarly, it was recently used to illustrate changes in land-based heat wave intensity in future scenarios in the Intergovernmental Panel on Climate Change (IPCC) AR6 synthesis report (*35*). However, extreme events, such as MHWs, can be described by multiple characteristics, such as frequency, duration, cumulative intensity, or the recurrence interval between succeeding MHWs (*36*). The definition of MHWs is currently an active area of debate, with practical importance for ecosystem applications (*37*). The analysis of maximum SST adds value in addition to these MHW metrics, as it is comparatively simple, independent of a reference state and threshold definition, and based on established statistical theory. Hence, it facilitates comparing simulated SST maxima to observations and eventually gives greater confidence in the robustness of climate model projections of MHWs. Future work could also use the GEV to estimate return levels as another metric of MHWs.

Our analysis suggests that CMIP6 models capture ocean maximum temperatures well on the whole. This comparison provides strong quantitative evidence that CMIP6 models are well suited to making reliable projections about the future characteristics of MHWs under continued climate change. While many studies have shown that the frequency of MHWs will increase in the future (*4*, *5*), our approach identifies regions where significant changes are expected for the ocean—i.e., where historical observations lie within the range of models’ historical simulations and where this model range shifts significantly in the future. In agreement with previous studies (*4*, *5*), our results indicate changes in the intensity of extreme SSTs with global warming. In our analysis, the change in the location parameter dominates the shifts in the GEV distribution, corresponding to significant increases in annual SST maxima in the Indian Ocean, most of the Pacific Ocean, most of the Atlantic Ocean south of ~40°N, and portions of the Southern Ocean, for both scenarios and both warming levels considered here. The increase in location parameter and annual SST maxima is mainly due to increasing mean SST, consistent with previous analyses identifying trends in mean SST as the main driver of increases in different MHW metrics (*4*, *12*).

Some authors have advocated for defining MHWs relative to a shifting-mean baseline (*38*), wherein the long-term ocean warming signal is effectively removed. However, shifting their temperature thresholds at the same rate as the long-term warming may not be feasible for organisms with limited adaptation capability, such as warm water corals (*9*). Besides the dominant effect from long-term warming, we find consistent but smaller increases in annual SST maxima from increases in seasonality in all analyzed CMIP6 models and both warming levels. Note that the approach presented here has the advantage of not requiring reference to a background state or fixed threshold.

Although maximum temperatures become significantly warmer over most of the ocean under a lower-emissions scenario, our results suggest that emissions reductions will substantially reduce the rate of increase in maximum temperatures, and likely therefore substantially reduce the harmful impacts of MHWs on ocean ecosystems.

## MATERIALS AND METHODS

### Observations

The observations we analyze are the 0.05° resolution, but regridded to 1°, satellite SST product from the European Space Agency (ESA) Climate Change Initiative (CCI) (available via https://surftemp.net/, downloaded on 10 June 2022) (*39*). Note that the citation (*39*) describes data up to 2016, but since the time of publication, the dataset has been extended to include the data from 2017 to 2020 that we also use. The dataset version used is CCI SST v2.1, which has also been corrected for desert dust–related biases (*40*). It includes 39 complete years (1982–2020) and uses purely satellite-based observations without explicitly blending in situ observations. This dataset is uniquely suited to our purposes because of its thorough validation and rigorous construction and because it provides depth-adjusted SSTs de-aliased with respect to the diurnal cycle for direct comparison with model SSTs (*39*). The data were regridded to 1° to facilitate comparison with the model realizations we were able to obtain (see below). In general, this product and resolution were both chosen because they make the comparison between observations and models as direct as possible. The regridding is performed by the surftemp.net tool provided by the data generators and incorporates the same assumptions and corrections used to generate the underlying dataset, detailed in (*39*). The notable aspect of these is that a 7-day/3° temporal/spatial decorrelation scale is assumed; these values are not known exactly, but their orders of magnitude are known given that the process to which they are related is the imperfectly accounted-for influence of the atmospheric state on the estimated SST (*39*).Given the small size of the errors accounted for by this factor, it is implausible that these time and spatial scale estimates affect our conclusions. Future work with higher-resolution models should explore how GEV parameters depend on the spatial scale considered. Note that all GEV parameters are dependent on the block size and relatedly the spatiotemporal scales considered. Therefore, our analysis focuses on the suitability of the GEV as a description of SST maxima and the correspondence between model- and observation-derived GEV parameters at a given scale, rather than the exact values of these parameters.

### Model output

The model output we use is daily mean SST (tos, in CMIP notation) output regridded to 1° resolution from the Earth system models that participated in the CMIP6 (*23*). We were able to obtain one realization of 14 different models, provided by 10 modeling centers (Table 1). We use the historical simulations over the 1850–2014 period and the future projections over 2015–2100 from the ScenarioMIP simulations (*41*), in particular the low-emissions high-mitigation scenario SSP1-2.6 and the high-emission low-mitigation scenario SSP5-8.5. We used the latter scenario simulations to determine the decades in which each model exceeds 2° and 3.2°C of global mean surface temperature change (i.e., warming averaged over both land and sea) since preindustrial (i.e., 1850–1900) for Fig. 3. The value of 3.2°C was chosen because all of the models used here reached at least this level of warming, i.e., it is the maximum SST increase common to all the models. The global warming levels (GWLs) of 2.0° and 3.2°C represent 40-year time periods in the model simulations centered on the years when global mean surface air temperature crosses the respective temperature values. For the 2.0°C GWL, these years range from 2023 to 2057 with a median of 2045, and for the 3.2°C GWL from 2044 to 2081 with a median of 2070. The 40-year periods centered on these years represent periods when temperature transiently crosses 2.0° and 3.2°C global warming. Hence, the ocean is not yet in equilibrium with the atmosphere, and SST is lower compared to when the ocean is in equilibrium with the atmosphere at the same GWLs (*42*, *43*). We also use a 30-member ensemble for the historical period of a 15th model from CMIP5, GFDL ESM2M-LE (*27*, *44*, *45*). To test for the influence of internal variability on the variations of the quantities in Table 1, we perform the same analyses reported in Table 1 for this single model ensemble.

### Statistical analysis

**Fig. 6.**
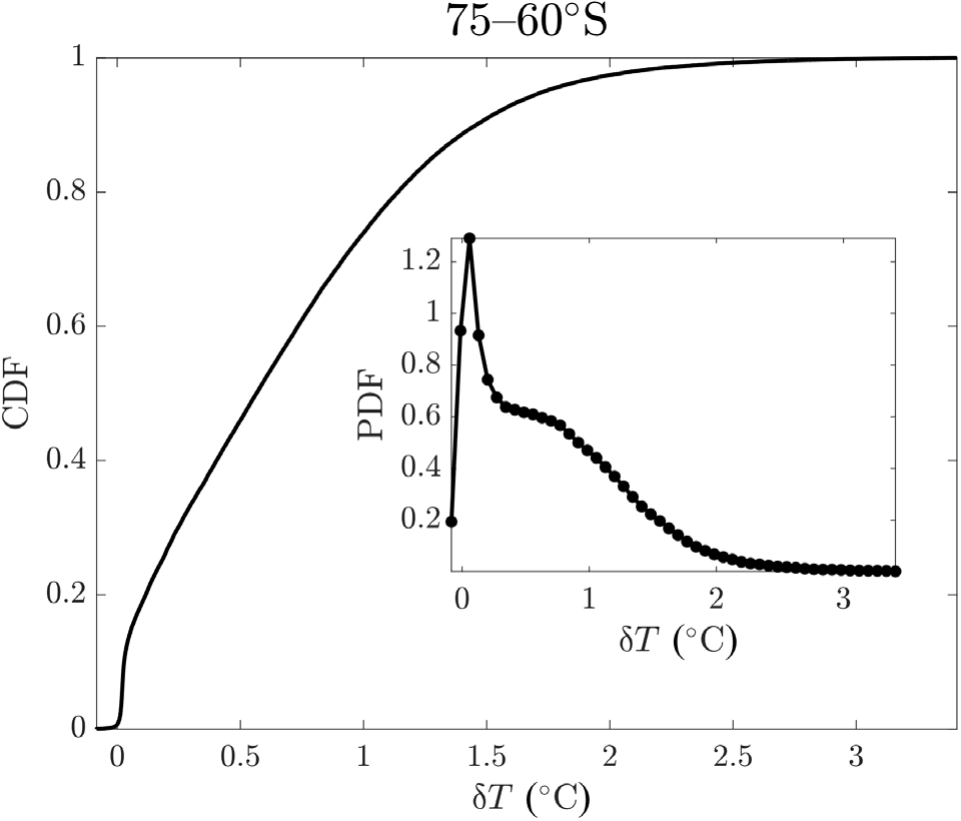
Empirical CDF and PDF for 75°S to 60°S.

Different approaches exist to define MHWs (*1*, *4*, *21*, *36*, *46*). Here, we consider exclusively the annual maximum of daily mean SST (unit of °C). We remove leap days from our analysis for simplicity. We only consider the latitudes 60°S to 70°N because latitudes poleward of these are affected by sea ice, which strongly alters both the characteristics and measurement of SST. Figure 6 illustrates this for 75°S to 60°S; the data appear to be a mixture of a GEV-like distribution and a narrow Gaussian distribution centered near zero. The latter of these is likely due to locations that are sea ice–covered throughout the year, substantially restricting their SST variation. Future work could address the high latitudes using a Gaussian-GEV mixture modeling approach; this analysis would be substantially more complicated than the analysis described here but would be valuable because high-latitude ecosystems may be particularly sensitive to the impacts of MHWs (*47*).

For both observations and model output, we consider both the “raw” maxima, i.e., the maximum daily mean SST in a given year, and the maximum “anomaly” (δ*T* in Figs. 1 and 6), from an interdecadal trend and a seasonal cycle. For the latter, we regress SST against a linear trend model with a categorical variable for each day of the year and then take the residuals from this regression for the anomalies. The trend that is subtracted from the daily mean SSTs by this calculation is the linear trend in daily mean SST over the full 39-year period. The seasonal cycle that is thereby subtracted from the daily mean SSTs is the average daily mean SST for each day of the year after the trend has been removed and over the same period. The anomaly is therefore relative to the average detrended SST value for a given day of the year. This allows us to simultaneously remove a linear interdecadal temperature trend and an annual seasonal cycle without making assumptions about the shape of the latter over the course of a year. Note, however, that this does assume a constant trend and seasonal cycle over time. Removing a seasonal cycle also means that maximum SST anomalies may occur at any point in the year, whereas maximum (raw) SSTs predominantly occur during times of year when average SSTs are already high. Note that the detrending of model output is performed separately for different 40-year periods, as is the removal of the seasonal cycle. SST trends over periods substantially longer than ~40 years are likely to be significantly nonlinear, and fitting such nonlinear trends with linear approximations can introduce artifacts into the residuals that would affect the GEV parameters and other metrics of interannual variability (*31*, *48*).

We then fit these raw maxima and maximum anomalies by a GEV distribution via maximum likelihood estimation using the “mle” (maximum likelihood estimate) function in Matlab 2021b. The extreme value theorem states that the GEV distribution is the only possible limit distribution of properly normalized maxima of a sequence of independent and identically distributed (i.i.d.) random variables. Here, we consider blocks of 1 year, i.e., annual maxima. Natural phenomena are rarely if ever truly i.i.d., but the GEV distribution holds and is applied broadly nonetheless (*13*). Autocorrelation does not bias tail estimation (*49*), and formally accounting for it in GEV parameter estimation is computationally intensive and does not significantly affect parameter values (*50*). Furthermore, in this case, only 17% of locations have significantly autocorrelated annual maximum daily mean temperatures at the 90% confidence level, with a median autocorrelation across grid cells of 0.12, so consideration of autocorrelation in our analysis is not justified and does not affect our conclusions.

**Fig. 7.**
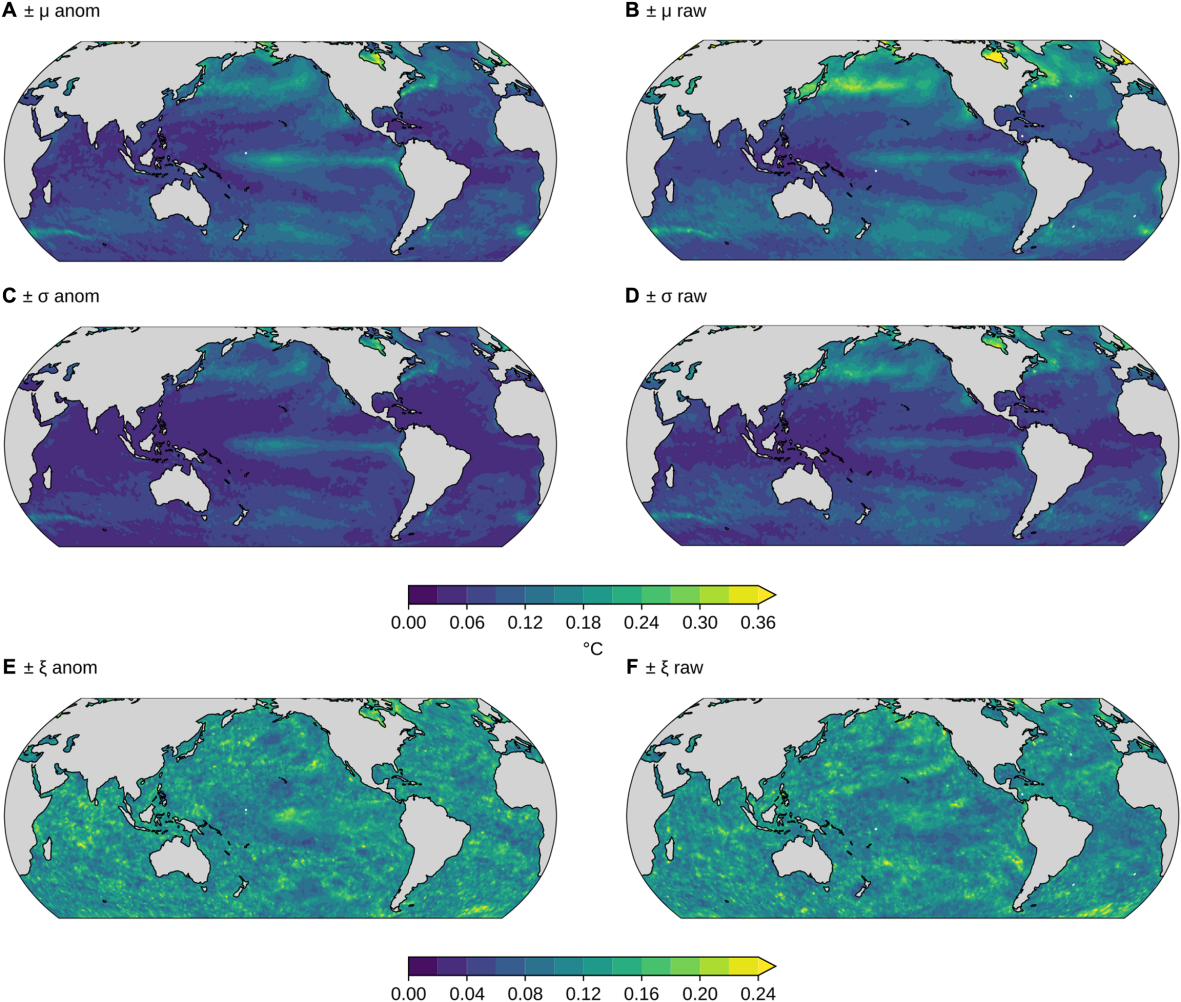
Observational GEV parameter uncertainties. One SD uncertainties of the maximum likelihood estimates for the anomaly (**A**) and raw (**B**) location parameter μ (°C), the anomaly (**C**) and raw (**D**) scale parameter σ (°C), and the anomaly (**E**) and raw (**F**) shape parameter ξ (∼) in the satellite SST observations. The color scale is the same for all subfigures and in the same units as each parameter. The uncertainties are due to the small sample size (*n* = 39) from which the GEV parameters are estimated for each location.

The GEV distribution has the form (*51*)

$$f\left(x;\mu ,\sigma ,\xi \right)=\frac{1}{\sigma }t{\left(x\right)}^{\xi +1}e^{-t\left(x\right)}$$

where *f*(·) is the probability density function and

$$t\left(x\right)=\left\{\begin{array}{cc} {\left[1+\xi \left(\frac{x-\mu }{\sigma }\right)\right]}^{-1/\xi } & \text{if}\;\xi \neq 0\\ e^{-\left(x-\mu \right)/\sigma } & \text{if}\;\xi =0 \end{array}\right.$$

where the range of *x* is such that [1 + ξ(*x* − μ)/σ] > 0, μ (°C) and σ (°C) are the location and scale parameters, and ξ is the parameter that controls the shape of the distribution. In our study, *x* is the annual maximum daily SST. A large positive ξ results in a heavy-tailed distribution, while a negative value of ξ results in a light-tailed distribution. The extent to which the empirical distribution of maxima deviates from the GEV is then determined by calculating the Kuiper statistic *V*, which is the maximum of the empirical minus hypothesized CDFs plus the maximum of the empirical minus hypothesized CDF, i.e.,

V=max[E(x)−H(x)]+max[H(x)−E(x)]

where *E*(*x*) is the empirical CDF of *x* and *H*(*x*) is the hypothesized empirical CDF of *x*. For a GEV distribution, *H*(*x*) has the form *H*(*x*) = *e*^−*t*(*x*)^ for the function *t*(*x*) above. This statistic is chosen over the more common Kolmogorov-Smirnov statistic *D* = max∣*E*(*x*) − *H*(*x*)∣ because it is equally sensitive for all values of the random variable *x* (*24*). (Repeating all analysis with *D* instead of *V* does not affect our conclusions.) The Kuiper statistic takes values in the range [0,2], with lower values indicating closer correspondence. We first fit the GEV of the maximum anomalies, pooled across both all years and all locations; the parameters and *V* value associated with this fit are given in Fig. 1. Values of *V* or other CDF statistics are difficult to interpret for large observational sample sizes because one cannot distinguish whether minute detected differences between empirical and hypothesized distributions are due to measurement errors versus process-relevant factors. We therefore not only rely on the quantitative value of *V* but also evaluate correspondence between the observations and the GEV visually in multiple ways in Fig. 1. Given the excellent correspondence seen in Fig. 1, we then fit the distribution of the 39 years of annual maximum temperatures (both raw and anomalies) at each location. The associated parameter values are given in Fig. 2. In Fig. 7, the standard (i.e., ±1 SD) uncertainties of the μ and σ values estimated for observations are shown; these are calculated by the Wald method using the approximate Hessian matrix at the maximum likelihood estimates to compute SEs. This method demonstrates that the estimation variability for the global parameter values is negligible. The same fitting procedure is then repeated both for globally pooled maximum anomalies and for local raw maxima and maximum anomalies for each model realization, both for the 39-year historical period matching the observations and for future periods (see below).

Figure 3 shows the model ensemble mean of the parameter changes from 1982–2020 to (A) 2061–2100 for SSP1-2.6, (B) 2061–2100 for SSP5-8.5, (C) the 40-year period centered on when 2°C warming is reached in each model in SSP5-8.5, and (D) the 40-year period centered on when 3.2°C warming is reached in each model in SSP5-8.5. The black stippling indicates regions where the 90% range (i.e., the 5th to 95th percentile) of the model ensemble distribution for each mapped quantity includes zero. This 90% range is estimated as the model ensemble mean plus or minus 1.645 times the model ensemble SD (n.b. 1.645 is the *z* score associated with the 95th percentile of a standard normal random variable). The pink stippling indicates regions where the 90% range of the model ensemble distribution for each mapped quantity in the 39-year historical period does not include the observational estimate of that quantity.

In Table 1, in the global section, the *V* and parameter values are given for each model realization by following the same procedure as in Fig. 1 but for the historical model output rather than the observations. In the anomalies and raw sections, the *r*^2^ values indicate the fraction of the variance accounted for in the observed parameters’ (spatial) distribution by the models’ parameters’ (spatial) distributions. *r*^2^ = 1 − RSS/TSS, where RSS is the residual sum of squares—here the residual being the difference in a given parameter’s values at each location for a given model versus the observations—and TSS is the total sum of squares for the observations. An *r*^2^ = 1 thus indicates an everywhere perfect correspondence between the observed and modeled values. The $\tilde{V}$ values indicate the median value of *V* across GEV fits to all locations. To contextualize the magnitude of these *V* values, we generate 10,000 sets of 39 draws each from a known GEV(0,1,0) distribution and fit each of these with a GEV exactly like we do the sets of annual maximum temperatures. The median *V* value for these sets is 0.14, which thus indicates high correspondence between the underlying and fitted distributions. Varying the GEV parameters within the range of the values found for SST maxima here does not change this result.

### Testing for nonstationarity

We tested for nonstationarity by repeating the analysis shown in Fig. 1 for the spatially pooled anomalies for individual years. Note that the raw SST data cannot be aggregated in space and fit with a GEV to test for nonstationarity in this way. We repeated this process both with globally pooled anomalies and with regionally pooled anomalies, defining regions corresponding to the equatorial and eastern tropical Pacific, the rest of the subtropics, and the subpolar regions poleward of 30 N/S. None of the parameters exhibited a significant trend in any region (bootstrap 90% confidence intervals of trends, estimated by linear regression of parameter estimates versus year, all included zero), indicating a lack of appreciable nonstationarity in these data. Note that the anomalies include a linear interdecadal trend, but μ could be nonstationary even for these detrended data if maximum SST values were increasing significantly faster or slower than annual mean SSTs. This does not wholly exclude the possibility of nonstationarity of course, given the small sample size of 39 years; a more thorough analysis of nonstationarity behavior is outside the scope of this manuscript but may be fruitful to pursue in particular with large model ensembles with many realizations using a single model.

### Testing for regional applicability

**Fig. 8.**
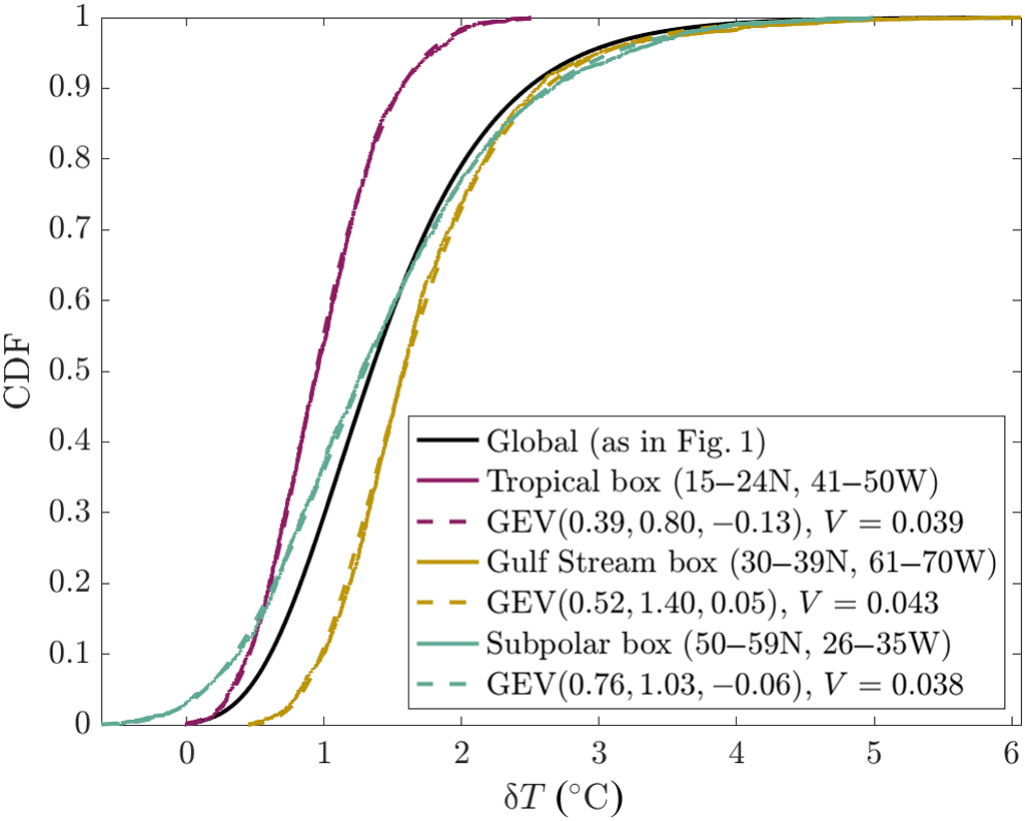
Empirical CDF for 10° × 10° boxes.

In addition to the local (1°) and global GEV fits described above, we perform an additional analysis to test whether the GEV is applicable to SST maxima at the regional scale. We define three 10^°^ × 10^°^ boxes in the North Atlantic—a tropical box at 15°N to 24°N, 41°W to 50°W; a Gulf Stream box at 30°N to 39°N, 61°W to 70°W; and a subpolar box at 50°N to 59°N, 26°W to 35°W. These boxes are defined so as to represent different dynamical regions. We then repeat the analysis from Fig. 1 on the subsets of the observations within each box. We expect the GEV to be applicable at this scale, with slightly larger *V* values due to having ∼500× smaller sample sizes and with parameters that vary between regions. The result of the regional analysis is shown in Fig. 8. As expected, we find that the GEV captures the distributions of SST maxima in these regions, with plausible variations in the distributions corresponding to each region.
